# Use of miRNAs as Biomarkers in Sepsis

**DOI:** 10.1155/2015/186716

**Published:** 2015-06-28

**Authors:** Raluca Dumache, Alexandru Florin Rogobete, Ovidiu Horea Bedreag, Mirela Sarandan, Alina Carmen Cradigati, Marius Papurica, Corina Maria Dumbuleu, Radu Nartita, Dorel Sandesc

**Affiliations:** ^1^Department of Forensic Medicine, “Victor Babes” University of Medicine and Pharmacy, 300041 Timisoara, Romania; ^2^Clinic of Anaesthesia and Intensive Care, Emergency County Hospital “Pius Brinzeu”, 300736 Timisoara, Romania; ^3^Faculty of Medicine, “Victor Babes” University of Medicine and Pharmacy, 300041 Timisoara, Romania; ^4^Faculty of Chemistry, Biology, and Geography, West University of Timisoara, 300115 Timisoara, Romania; ^5^Clinic of Anaesthesia and Intensive Care “Casa Austria”, Emergency County Hospital “Pius Brinzeu”, 300736 Timisoara, Romania

## Abstract

Sepsis is one of the most common causes of death in critical patients. Severe generalized inflammation, infections, and severe physiological imbalances significantly decrease the survival rate with more than 50%. Moreover, monitoring, evaluation, and therapy management often become extremely difficult for the clinician in this type of patients. Current methods of diagnosing sepsis vary based especially on the determination of biochemical-humoral markers, such as cytokines, components of the complement, and proinflammatory and anti-inflammatory compounds. Recent studies highlight the use of new biomarkers for sepsis, namely, miRNAs. miRNAs belong to a class of small, noncoding RNAs with an approximate content of 19–23 nucleotides. Following biochemical and physiological imbalances, the expression of miRNAs in blood or other body fluids changes significantly. Moreover, its stability, specificity, and selectivity make miRNAs ideal candidates for sepsis biomarkers. In conclusion, we can affirm that stable species of circulating miRNAs represent potential biomarkers for monitoring the evolution of sepsis.

## 1. Introduction

Sepsis is one of the most common causes of death in the hospitalized patients in the intensive care unit [1]. It represents a clinical syndrome resulting from the interaction between the infective pathogen and systemic inflammatory response. In recent years, sepsis remains a challenge for the clinician, especially in terms of monitoring the efficacy of treatment [2]. The increased percentage of patients suffering from sepsis imposed developing new protocols consisting in rapid, inexpensive methods with high specificity and selectivity for evaluation and monitoring treatment. At the moment there are a number of biomarkers for sepsis, mainly used in clinical laboratory analysis. The most used biomarkers for sepsis are procalcitonin (PCT) [3], C-reactive protein (CRP), and interleukin 6 (IL-6) [2]. The problem with these biomarkers is given by their low selectivity and specificity. Recent studies call into question the use of new biomarkers for sepsis, such as miRNAs [4]. The properties that these species have in addition to the conventional biomarkers are their higher stability, selectivity, and specificity [5]. In the present work, we want to present and highlight the possibility of using miRNA species as biomarkers for diagnosis, monitoring, and guiding of the therapy in patients with sepsis.

## 2. Structural and Biochemical Aspects of miRNAs 

**Figure 1:**
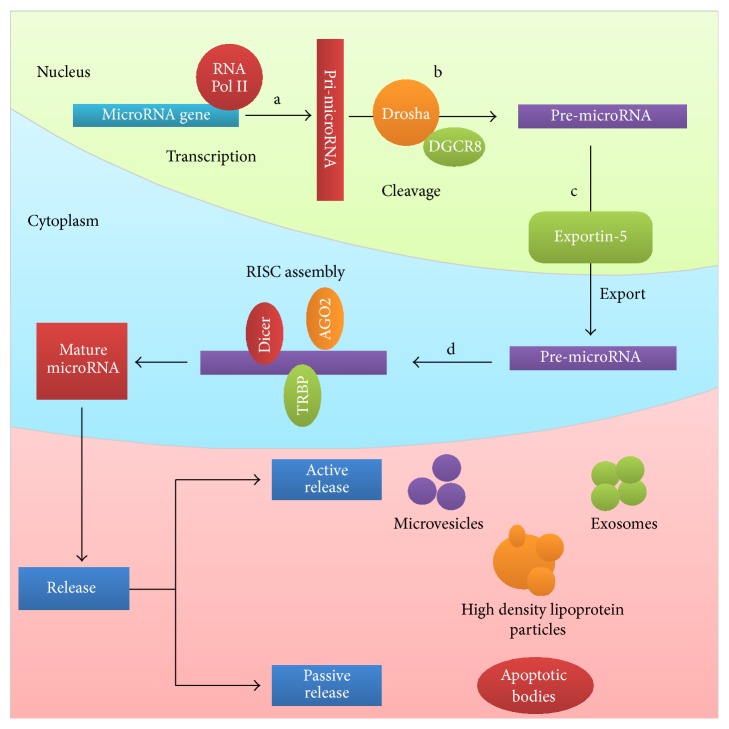
miRNA biogenesis mechanism. miRNA synthesis begins with RNA polymerase II action on protein coding genes. (a) Through the transformation phenomenon of the miRNAs genes, pri-miRNA is forming. (b) By the action of RNase III endonuclease (Drosha) and of the DiGeorge Syndrome Critical Region 8 (DGCR8) cofactor, the pre-miRNA is forming. (c) Through transporting protein Exportin-5, pre-miRNA is transferred from the nucleus into the cytoplasm. (d) In the cytoplasm pre-miRNA is attacked by second RNase III endonuclease (Dicer) and transactivator RNA binding protein forming mature miRNA (double-stranded) and miRNA^*∗*^ (passenger strand). In what follows, mature miRNA induced silencing is taken in complex (RISC). RISC complex contains mature miRNA and protein Argonaute 2 (AGO) that confers increased stability of the complex. After this, miRNAs are released from the cell by two mechanisms: active release (microvesicles, exosomes, and high density lipoprotein particles) and passive release (apoptotic bodies).

miRNAs are noncoding RNA generally formed of 19–24 nucleotides [6]. The first miRNA species was discovered since 1993 in* Caenorhabditis elegans* and it was called lin-4 [7]. miRNAs synthesis occurs in the cell nucleus through the action of RNA polymerase II on miRNA genes. Through transcription, pre-miRNA species are obtained. Through the action of RNase III endonuclease, called Drosha, pre-miRNA is obtained. In order for the transformation to take place, Drosha requires the cofactor DiGeorge Syndrome Critical Region 8 (DGCR8) [8–10]. After the formation of pre-miRNA in the nucleus, this species is transferred into the cytoplasm through Exportin-5. Once in the cell cytoplasm, pre-miRNA species is cleaved by a second RNase III endonuclease, called Dicer, along with transactivator RNA binding protein (TRBP) to form mature miRNA (double-stranded) and miRNA^*∗*^ (passenger strand) [11]. Eventually miRNA will be degraded by the Argonaute protein. The next step in biogenesis of miRNAs is introducing the mature species in the RNA induced silencing complex (RISC). The miRNAs are specifically released by cells under certain conditions of stress. The release mechanisms of miRNAs are passive release when cell death occurs (apoptotic bodies) and active release when cellular secretions occur (exosomes, ribonucleoprotein complexes, high density lipoproteins, and microvesicles) [12, 13]. In Figure 1 is presented the biogenesis mechanism of miRNAs.

## 3. The Use of miRNAs as Biomarkers in Clinical Diagnosis

In order for a macromolecule or a biochemical species to be used as a biomarker, it must meet certain properties. Regardless of the area of use, biomarkers should be accessible by noninvasive, cheap, and fast methods. Another important aspect is given by the specificity for a particular tissue or for a specific pathology/injury. Regarding the specificity, selectivity, and high stability of miRNAs, this makes them ideal biomarkers in various fields such as forensics, clinical diagnostic analysis of medical research [14].

A significant number of miRNAs are found at intracellular level. However, many studies report the existence of a significant number of miRNAs outside the cell, called circulating miRNAs. Extracellular miRNAs have been identified in several biological fluids, such as blood, urine, saliva, peritoneal fluid, amniotic fluid, bronchial lavage, cerebrospinal fluid, and tears [15–17].

Basic features of extracellular miRNAs are represented by high stability and specificity. Although their stability in the extracellular environment is high, most often their stability is increased by encapsulation in lipid vesicles or by forming complexes with various proteins in order to protect them against denaturation. Thus, many miRNAs are found in biological fluids as exosomes, microvesicles, or high density lipoprotein particles [18]. The body fluids are the most accessible biological samples, ideal for the analysis of specific biomarkers. Recent studies report the presence of an increased number of miRNAs specific to each type of biological fluid.  Table 1 summarizes the specificity for different types of fluids. Therefore, body fluids are the most accessible biological samples ideal for the analysis of impressive number of biomarkers specific. miRNAs can be detected by DNA microarray [19], quantitative reverse transcription PCR [20], or RNA sequencing [21]. A number of studies talk about the possibility of correlating the expression of miRNAs with a series of pathologies. Thus, the question of using miRNAs as biomarkers for a number of physiological imbalances and diseases was raised. At the moment, different types of miRNAs have been correlated with cardiovascular disease, various cancers, pathophysiological dysfunction, poisoning with various substances, diseases of the central nervous system, metabolic disorders, immunological disorders, infections, and posttraumatic disorders [5, 22–24].

In recent years there has been a very intense research regarding cancer diagnosis through miRNAs. Numerous studies have identified a series of specific miRNAs for each type of cancer in part. Mitchell et al. [25] identified six miRNAs that could serve as biomarkers in prostate cancer diagnosis by noninvasive methods: miRNA-100, miRNA-125b, miRNA-141, miRNA-143, miRNA-205, and miRNA-296 [25]. Ho et al. [26] studied specific miRNA biomarkers for pancreatic cancer and identified the expression of miRNA-210 as a potential candidate [26]. Wang et al. [27] in a similar study identified four miRNAs that could serve as biomarkers in the diagnosis of pancreatic cancer: miRNA-21, miRNA-210, miRNA-155, and miRNA-196a [27]. Lin et al. [28] reported a total of five miRNAs whose expression could serve as a biomarker in the diagnosis of liver cancer: miRNA-15b, miRNA-1975, miRNA-199a-3p, miRNA-199b-3p, and miRNA-421 [28]. Also, a number of miRNAs that can serve as diagnostic biomarkers for colorectal cancer have been identified [29]. Since the death rate from colorectal cancer can be reduced by applying the diagnosis and treatment in the initial stage, several groups of researchers have studied the expression of miRNAs in patients with this condition. Wang et al. [30] showed an increased expression of miRNA-21 and let-7g and also a decreased expression of miRNA-31, miRNA-181b, miRNA-92a, and miRNA-203 in patients with colorectal cancer [30]. Yang et al. [31] also identified miRNA-29c in this type of patients [31]. Tsujiura et al. [32] identified in patients with gastric cancer an increased expression of miRNA-17-5p, miRNA-21, miRNA-106a, miRNA-106b, miRNA-17, and let-7a [32]. Also, current studies confirm the existence of significant correlations between the expression of different miRNAs and a number of cancers. According to the literature, for each type of cancer there might be a specific miRNA that could serve in the future as a noninvasive diagnosis tool [33].

miRNAs can serve to diagnose not only cancers but also other pathologies responsible for a high death rate worldwide. For example, cardiovascular dysfunction kills annually a high percentage of people of all ages worldwide. Diagnostic methods are often expensive and invasive and with low specificity. Using the expression of miRNAs to obtain a differential diagnosis in cardiovascular pathologies is the main subject of study for many research groups [34]. Stather et al. [35] have studied the expression of miRNAs in patients with peripheral arterial disease. The study revealed a number of specific miRNAs for this condition: miRNA-15b, miRNA-16, miRNA-20b, miRNA-25, miRNA-26b, miRNA-27b, miRNA-28-5p, miRNA-126, miRNA-195, miRNA-335, and miRNA-363 [35]. Jansen et al. [36] also revealed a number of miRNAs whose expression was altered in patients with stable coronary artery disease: miRNA-126, miRNA-222, miRNA-21, miRNA-20a, miRNA-27a, miRNA-92a, miRNA-130, miRNA-199a, miRNA-17, miRNA-222, miRNA-21, miRNA-20a, miRNA-27a, miRNA-130, miRNA-92a, and miRNA-17. Moreover, they observed that low concentration of miRNA-126 and miRNA-199a may be correlated with a decreased risk of cardiovascular events [36]. Other cardiovascular pathologies, including arterial hypertension, myocardial infarction, and ischemia, will lead directly to the release of specific biomarkers. Leptidis et al. [37] reported and validated the existence of a series of specific miRNAs for the myocardial infarction: miR-24, miR-125b, miR-214, and miR-195 [37].

Regarding the neurodegenerative diseases such as Alzheimer's disease and Parkinson's disease, many miRNAs have been identified that can be used in the diagnosis of these disorders. Wang et al. [38] studied the expression of miRNAs in the patients with Alzheimer's disease, proving that miRNA-146 is upregulated, unlike in healthy patients [38]. Tan et al. [39] in a similar study observed and reported that miRNA-125b and miRNA-181c are downregulated, while miRNA-9 is upregulated [39]. Zhao et al. [40], in the study regarding the expression of miRNAs in Parkinson's disease, reported low levels of miRNA-133b in these patients [40]. In a similar study, Alieva et al. [41] reported increased levels of the following miRNAs: miRNA-7, miRNA-9-5p, miRNA-9-3p, miRNA-129, and miRNA-132.

Both for the emergency units and for the intensive care units, critical patient is a challenge. Corroborating the acute and chronic pathologies, survival rate drops dramatically. Multiple trauma is most often fatal for this type of patients [42]. Spinal cord along with traumatic brain injury is one of the most serious injuries, with a high mortality rate [43, 44]. Expression of miRNAs was studied in severe trauma by different research groups. Izumi et al. [45] studied the expression of miRNA in experimental models with spinal cord injury and reported abnormal expression of miRNA-233 12 hours after injury. In the case of traumatic brain injury (TBI), Lei et al. [46] reported an increased expression of miRNA-21 [46]. One of the severe consequences of posttraumatic injury is represented by severe systemic inflammation, often accompanied by systemic generalized infections. A significant percentage of patients with sepsis develop multiple organ failure. In this case the mortality reaches a dramatic level up to 70% [47]. Numerous studies report the existence of a high level of miRNAs in patients with sepsis, leading to the introduction of possible new biomarkers in monitoring sepsis in such patients [19, 48, 49].

## 4. Circulating miRNAs as Biomarker for Sepsis

Sepsis is a potentially life-threatening complication of an infection. Sepsis occurs when chemicals released into the bloodstream to fight the infection trigger inflammatory responses throughout the body [1, 50]. This inflammation can trigger a cascade of changes that can damage multiple organ systems, causing them to fail. Sepsis is divided into three categories according to the nature, quantity, and the germs virulence: moderate sepsis, severe sepsis, and septic shock [51, 52]. In case of septic shock, the volemic management becomes challenging in most cases due to lack of response to fluid loading, imposing the implementation of pharmacological support for maintaining physiological parameters. The inflammatory is determined mostly by a series of inflammatory mediators. By their synergistic or antagonistic action, both beneficial and adverse effects can occur, which can lead to complete damage of the cell [53–55]. The inflammatory cascade is triggered or augmented by the presence of microorganisms and by toxins. Some microorganisms produce exotoxin (staphylococci and streptococci), others endotoxin (*E. coli*), and others both exotoxin and endotoxin (*Pseudomonas*) [56]. The endotoxin is the most involved in the septic shock, mostly due to its biochemical structure (macromolecular complex glucose lipid protein included in the bacterial cell wall) [57].

Complement activation (C) usually precedes hemodynamic disturbances in serious infections. One of the main roles of C is to enable leukocytes to adhere to the endothelium and to release large amounts of inflammatory mediators. Moreover, it interferes with the biochemical function of some enzymes, increasing capillary permeability [57, 58].

The most important inflammatory mediators are cytokines. Their synthesis in sepsis is due to the interaction between a fraction of a lipopolysaccharide (LPS) and a protein normally present in the human body, respectively, lipopolysaccharide binding protein (LBP), with the CD14 receptor on the surface of macrophages [50]. Also, sepsis implies activation of the coagulation cascade and synthesis of other mediators such as hormones, histamine, arachidonic acid derivatives, and chemokines [2, 59, 60].

During sepsis, the inflammatory response is mediated by the activation of toll-like receptor (TLR) and also by downregulation of NF-KB pathway within the macrophages and monocytes [61]. Tsujimoto et al. [62] demonstrated that TLR are also involved in the development of the septic shock. Presently, 10 types of TLR were identified. TLR1, TLR2, TLR3, TLR4, TLR5, and TLR6 are stimulated by some proteins and lipids from the microbial walls. On the other hand, due to their localization into the endoplasmic reticulum, endolysosomes, lysosomes, and endosomes, TLR7, TLR8, and TLR9 present the property of recognizing the microbial nucleic acids [63]. TLR-NF-KB inflammatory response is also involved in the process of sepsis. Due to this fact, the use of corticosteroids, antagonists of tumor necrosis factor (TNF), and antagonists of interleukin 1 receptor does not have good results in treating sepsis [61, 62, 64, 65].

A number of analytical diagnostic methods have been developed over time in order to help monitor and evaluate patients with sepsis. The most common used biomarkers in the diagnosis and evaluation of sepsis are as follows: interleukin 1 (IL-1), interleukin 2 (IL-2), interleukin 6 (IL-6), interleukin 12 (IL-12), interleukin 8 (IL-8), interleukin 4 (IL-4), interleukin 10 (IL-10), interleukin 17 (IL-17), interleukin 13 (IL-13), tumor necrosis factor alpha (TNF-alpha), interferon gamma (INF-gamma), transforming growth factor beta (TGF-beta), procalcitonin (PCT), N-terminal C natriuretic peptide (NT-CNP), C-reactive proteins (CRP), granulocytes and monocytes colony stimulating factor (GM-CSF), leukotrienes, prostaglandins and thromboxane, or components of the complement (C3a and C5a) [2, 3, 66].

For a faster and cheaper diagnosis, in the recent years the researchers have tried new methods of analysis, with the most intensively studied being circulating miRNAs. Recent studies have revealed the presence of a relatively high number of miRNAs whose expression can be correlated with sepsis [67]. Puskarich et al. [68] studied the expression of miRNA-146a, miRNA-223, and miRNA-150. They reported a correlation between the expression of these three miRNAs and sepsis. Moreover, their study shows a direct correlation between the expression of miRNA-150 and a high mortality rate [68]. Vasilescu et al. [69] have studied the expression of miRNAs in patients with sepsis. They report a decrease in the expression of miRNA-150 and miRNA-342-5p in patients with sepsis as opposed to the healthy patients. Moreover, the expression of miRNA-486 and miRNA-182 was much higher in patients with sepsis versus healthy patients, according to the study conducted by Vasilescu and collaborators [69]. Roderburg et al. [14] in a similar study reported an increased expression of miRNA-150 in patients with sepsis. Wang et al. [70] have also studied miRNAs expression in critical patients with sepsis. The study concluded that the expression of miRNA-223 and miRNA-146a is lower in the group of patients with sepsis. In a similar study, Wang et al. [71] confirm these results by highlighting an increased expression of miRNA-146a in healthy patients.

However, there are studies suggesting that miRNA-223 cannot be used as a biomarker for sepsis. Benz et al. [72] in a similar study demonstrate that there is no difference in miRNA-233 levels in patients with sepsis and healthy patients [72]. Another group of miRNAs that can be used as biomarkers for sepsis belongs to the family of miRNA-4772. Ma et al. [73] studied miRNAs from miRNA-4772 family, emphasizing that three of them are relevant to the diagnosing of sepsis. Thus, in the study, they report an increased expression of miRNA-4772-5p-iso, miRNA-4772-5p, and miRNA-4772-3p in patients with sepsis [73].

Huang et al. [74] identified ten miRNAs that can serve as biomarker for sepsis: let-7b, miRNA-15b, miRNA-16, miRNA-210, miRNA-324-3p, miRNA-484, miRNA-486-5p, miRNA-340, and miRNA-324-3p [74]. Wang et al. [54], in a study on the expression of miRNAs conducted on 232 patients, demonstrated that the expression of miRNA-122 is significantly altered compared to healthy patients [54]. In Table 2 are summarized the expressions of miRNAs which may have significant importance in the diagnosis of sepsis.

A promising method is the use of specific miRNAs to detect microbiological species instead of blood cultures. This can be possible by detecting changes in the expression of miRNAs made by microorganisms. The specificity and selectivity of the method can be increased by detecting the changes in the expression of miRNAs in various bacterial infections [75].

Recent studies report an altered expression of miRNA-146 and miRNA-155 in case of* Helicobacter pylori *infection [76],* Listeria monocytogenes* [77],* Mycobacterium tuberculosis* [78], and* Salmonella enterica* [79].

In case of* Staphylococcus aureus, *four specific miRNAs were identified: bta-miRNA-2229, miRNA-499, miRNA-23a, and miRNA99b [80]. Zheng studied the expression of miRNAs in case of* Brucella melitensis*, identifying the presence of miRNA-92a, miRNA-93, miRNA-181b, and miRNA-1981 [81].

Infections generated by* Pseudomonas aeruginosa* also modify the expression of miRNAs, especially miRNA-302b [82] and miRNA-233 [83].

How et al. studied the expression of miRNAs in patients with Gram-negative bacilli induced urosepsis. They reported a decreased expression of miRNA-150 (*P* < 0.001) and let-7a (*P* < 0.001) compared with healthy patients [84]. Finally, we can say that the use of miRNAs as diagnostic biomarkers may represent a new perspective in the differential diagnosis between Gram-positive and Gram-negative bacteria.

## 5. Conclusions

Circulating miRNAs become more widely studied and more used as a biomarker for the diagnosis of a broad spectrum of physiological, metabolic, and biochemical dysfunctions. Using miRNAs as circulating biomarker for sepsis is still in its infancy and additional studies are required to increase the specificity and selectivity of this method. However, at the moment, a high number miRNAs have been validated as specific for sepsis. Strengthening a broader range of specific miRNAs for sepsis is required. In conclusion, we can affirm that it is necessary to improve detection and validation methods of specific miRNAs for sepsis.

## Figures and Tables

**Table 1:** miRNAs expression in body fluids.

miRNAs	Body fluid	Reference(s)
miRNA-135a^*∗*^; miRNA-139-39; miRNA-182; miRNA-224; miRNA-299-5p; miRNA-330-5p; miRNA-369-3p; miRNA-373; miRNA-483-3p; miRNA-483-3p; miRNA-508-3p; miRNA-518f^*∗*^; miRNA-519d; miRNA-551b; miRNA-801	Plasma	[69, 85–87]

miRNA-16; miRNA-20a; miRNA-106a; miRNA-126; miRNA-150; miRNA-185; miRNA-451	Venous blood	[85, 86, 88–92]

miRNA-26a; miRNA-96^*∗*^; miRNA-135b; miRNA-141; miRNA-145^*∗*^; miRNA-182^*∗*^; miRNA-200c; miRNA-203; miRNA-205; miRNA-381; miRNA-622; miRNA-658; miRNA-1228	Saliva	[15, 85, 86, 88, 89, 91]

miRNA-515-3p; miRNA-335; miRNA-873; miRNA-616; miRNA-134; miRNA-923; miRNA-101; miRNA-589; miRNA-545; miRNA-377; miRNA-890; miRNA-505; miRNA-302d	Urine	[85, 86, 93]

miRNA-10a; miRNA-10b; miRNA-17; miRNA-135a; miRNA-135b; miRNA-340; miRNA-380; miRNA-507; miRNA-644; miRNA-891a; miRNA-943	Semen	[86, 88, 89, 94]

miRNA-124a; miRNA-372; miRNA-617	Vaginal secretions	[86, 89]

miRNA-144; miRNA-185; miRNA-412; miRNA-451	Menstrual blood	[86, 89, 95]

miRNA-10a; miRNA-28-5p; miRNA-150; miRNA-193b; miRNA-217; miRNA-924	Breast milk	[86, 89, 96, 97]

miRNA-29-b-1; miRNA-129; miRNA-223; miRNA-627; miRNA-223; miRNA-583	Peritoneal fluid	[85, 86]

miRNA-577	Cerebrospinal fluid	[85, 86, 94]

miRNA-637	Tears	[85, 86, 89]

**Table 2:** miRNAs expression in sepsis.

miRNAs	Expression	Reference(s)
miRNA-150	Some studies report low plasma concentrations in patients with sepsis. However, similar studies reveal no statistically significant differences between patients with and without sepsis. Finally, low levels of miRNA-150 are associated with poor prognosis in critical ill patients	[14, 64]

miRNA-223; miRNA-146a	Downregulating in patients with sepsis	[70, 98]

miRNA-133a	Upregulating in patients with sepsis	[67]

miRNA-181b	Downregulating in patients with sepsis	[9]

miRNA-146a	Upregulating in healthy patients, as opposed to the group of patients with sepsis	[71]

miRNA-16	Upregulating in patients with sepsis	[54, 98]

miRNA-574-5p	Upregulating in patients with sepsis	[99]

miRNA-4772-3p; miRNA-4772-5p; miRNA-4772-5p-iso	Upregulating in patients with sepsis	[73]

miRNA-122	Statistically significant differences regarding the miRNA in patients with sepsis as opposed to the healthy patients	[54]
